# Self-Administered Outpatient Antimicrobial Infusion by Uninsured Patients Discharged from a Safety-Net Hospital: A Propensity-Score-Balanced Retrospective Cohort Study

**DOI:** 10.1371/journal.pmed.1001922

**Published:** 2015-12-15

**Authors:** Kavita P. Bhavan, L. Steven Brown, Robert W. Haley

**Affiliations:** 1 Division of Infectious Diseases, Department of Internal Medicine, University of Texas Medical Center, Dallas, Texas, United States of America; 2 Parkland Health and Hospital System, Dallas, Texas, United States of America; 3 Division of Epidemiology, Department of Internal Medicine, University of Texas Medical Center, Dallas, Texas, United States of America; University of Oxford, UNITED KINGDOM

## Abstract

**Background:**

Outpatient parenteral antimicrobial therapy (OPAT) is accepted as safe and effective for medically stable patients to complete intravenous (IV) antibiotics in an outpatient setting. Since, however, uninsured patients in the United States generally cannot afford OPAT, safety-net hospitals are often burdened with long hospitalizations purely to infuse antibiotics, occupying beds that could be used for patients requiring more intensive services. OPAT is generally delivered in one of four settings: infusion centers, nursing homes, at home with skilled nursing assistance, or at home with self-administered therapy. The first three—termed healthcare-administered OPAT (H-OPAT)—are most commonly used in the United States by patients with insurance funding. The fourth—self-administered OPAT (S-OPAT)—is relatively uncommon, with the few published studies having been conducted in the United Kingdom. With multidisciplinary planning, we established an S-OPAT clinic in 2009 to shift care of selected uninsured patients safely to self-administration of their IV antibiotics at home. We undertook this study to determine whether the low-income mostly non-English-speaking patients in our S-OPAT program could administer their own IV antimicrobials at home with outcomes as good as, or better than, those receiving H-OPAT.

**Methods and Findings:**

Parkland Hospital is a safety-net hospital serving Dallas County, Texas. From 1 January 2009 to 14 October 2013, all uninsured patients meeting criteria were enrolled in S-OPAT, while insured patients were discharged to H-OPAT settings. The S-OPAT patients were trained through multilingual instruction to self-administer IV antimicrobials by gravity, tested for competency before discharge, and thereafter followed at designated intervals in the S-OPAT outpatient clinic for IV access care, laboratory monitoring, and physician follow-up. The primary outcome was 30-d all-cause readmission, and the secondary outcome was 1-y all-cause mortality. The study was adequately powered for readmission but not for mortality. Clinical, sociodemographic, and outcome data were collected from the Parkland Hospital electronic medical records and the US census, constituting a historical prospective cohort study. We used multivariable logistic regression to develop a propensity score predicting S-OPAT versus H-OPAT group membership from covariates. We then estimated the effect of S-OPAT versus H-OPAT on the two outcomes using multivariable proportional hazards regression, controlling for selection bias and confounding with the propensity score and covariates.

Of the 1,168 patients discharged to receive OPAT, 944 (81%) were managed in the S-OPAT program and 224 (19%) by H-OPAT services. In multivariable proportional hazards regression models controlling for confounding and selection bias, the 30-d readmission rate was 47% lower in the S-OPAT group (adjusted hazard ratio [aHR], 0.53; 95% CI 0.35–0.81; *p* = 0.003), and the 1-y mortality rate did not differ significantly between the groups (aHR, 0.86; 95% CI 0.37–2.00; *p* = 0.73). The S-OPAT program shifted a median 26 d of inpatient infusion per patient to the outpatient setting, avoiding 27,666 inpatient days. The main limitation of this observational study—the potential bias from the difference in healthcare funding status of the groups—was addressed by propensity score modeling.

**Conclusions:**

S-OPAT was associated with similar or better clinical outcomes than H-OPAT. S-OPAT may be an acceptable model of treatment for uninsured, medically stable patients to complete extended courses of IV antimicrobials at home.

## Abstract 2

In a propensity score-balanced retrospective cohort study, Kavita Bhavan and colleagues compare health outcomes for patients undergoing self-administered versus healthcare-administered outpatient parenteral antimicrobial therapy.

## Editors' Summary

**Background:**

Patients sometimes need lengthy courses of antimicrobial agents to treat life-threatening infections. For example, patients who develop endocarditis (an infection of the inner lining of the heart usually caused by bacteria entering the blood and traveling to the heart) need to be given antimicrobial drugs for up to six weeks. Initially, these patients require intensive diagnostic and therapeutic care in the hospital. But once the antimicrobial treatment starts to work, most patients only need regular intravenous antimicrobial infusions. Patients who stay in the hospital to receive this low intensity care occupy beds that could be used for patients requiring more intensive care. Moreover, they are at risk of catching a hospital-acquired, antibiotic-resistant infection. For these reasons, and because long-term administration of antimicrobial agents in the hospital is costly, outpatient parenteral (injected or infused) antimicrobial therapy (OPAT) is increasingly being used as a safe and effective way for medically stable patients to complete a course of intravenous antibiotics outside the hospital.

**Why Was This Study Done?:**

In the US, OPAT is usually delivered in infusion centers, in nursing homes, or at home by visiting nurses. But healthcare-administered OPAT (H-OPAT) is available only to insured patients (in the US, medical insurance provided by employers or by the government-run Medicare and Medicaid programs funds healthcare). Uninsured people cannot usually afford H-OPAT and have to stay in safety-net hospitals (public hospitals that provide care to low-income, uninsured populations) for intravenous antibiotic treatment. In this propensity-score-balanced retrospective cohort study, the researchers investigate whether uninsured patients discharged from a safety-net hospital in Texas to self-administer OPAT at home (S-OPAT) can achieve outcomes as good as or better than those achieved by patients receiving H-OPAT. A retrospective cohort study compares recorded clinical outcomes in groups of patients who received different treatments. Because the patients were not chosen at random, such studies are subject to selection bias and confounding. Propensity score balancing is used to control for selection bias—the possibility that some members of the population are less likely to be included in a study than others. Adjustment for covariates (patient characteristics that may affect the outcome under study) is used to control for confounding—the possibility that unknown characteristics shared by patients with a specific outcome, rather than any treatment, may be responsible for that outcome.

**What Did the Researchers Do and Find?:**

Between 2010 and 2013, 994 uninsured patients were enrolled in the hospital’s S-OPAT program, and 224 insured patients were discharged to an H-OPAT program. Patients in the S-OPAT group were trained to self-administer intravenous antimicrobials, tested for their ability to treat themselves before discharge, and then monitored by weekly visits to the S-OPAT outpatient clinic. The researchers estimated the effect of S-OPAT versus H-OPAT on 30-day all-cause readmission and one-year all-cause mortality (the primary and secondary outcomes, respectively) after adjusting for covariates and controlling for selection bias with a propensity score developed using baseline clinical and sociodemographic information collected from the patients. The 30-day readmission rate was 47% lower in the S-OPAT group than in the H-OPAT group (a significant result unlikely to have arisen by chance), and the one-year mortality rate did not differ significantly between the two groups. Notably, because the S-OPAT program resulted in patients spending fewer days having inpatient infusions, 27,666 inpatient days were avoided over the study period.

**What Do These Findings Mean?:**

These findings indicate that, after adjusting for preexisting differences between those patients receiving S-OPAT and those receiving H-OPAT and for potential confounders, the risk of readmission within 30 days of discharge was lower in the S-OPAT group than in the H-OPAT group and the risk of dying within one year of hospital discharge did not differ significantly between the two groups (the study did not include enough participants to detect any subtle difference that might have existed for this end point). Thus, S-OPAT was associated with similar or better outcomes than H-OPAT. Note that there may be residual selection bias and confounding by characteristics not included in the propensity score. This study did not address whether S-OPAT actually improves outcomes for patients compared with H-OPAT; a randomized controlled trial in which patients are randomly assigned to receive the two treatments is needed to do this. Nevertheless, these findings suggest that S-OPAT might make it possible for uninsured, medically stable patients to have extended courses of intravenous antimicrobials at home rather than remaining in the hospital until their treatment is complete.

**Additional Information:**

This list of resources contains links that can be accessed when viewing the PDF on a device or via the online version of the article at http://dx.doi.org/10.1371/journal.pmed.1001922.

- The UK National Health Service Choices website provides basic information about the use of antibiotics, including information about when intravenous antibiotics are needed and about endocarditis
- The US National Heart, Lung, and Blood Institute also provides information about endocarditis and its treatment
- The Infectious Diseases Society of America provides clinical guidelines for the use of OPAT
- The OPAT Initiative of the British Society for Antimicrobial Chemotherapy is a multi-stakeholder project that supports the establishment of standardized OPAT services throughout the UK; it also provides guidelines for the use of OPAT
- Wikipedia has a page on propensity score matching (note that Wikipedia is a free online encyclopedia that anyone can edit; available in several languages)

## Introduction

A substantial source of hospital costs is the long-term administration of antimicrobial agents to patients with serious or life-threatening infections, such as osteomyelitis, endocarditis, and staphylococcal bacteremia [1–3]. A recent study estimated the number of endocarditis cases hospitalized in US hospitals in 2009 at 43,419, with an average stay of 15.3 d, generating average hospital charges of US$122,204 per case (estimated to be US$150,000 in 2014) [1]. Typically, patients with infections requiring long-term antibiotics receive intensive diagnostic and therapeutic services in the first several hospital days but thereafter remain in the hospital only to receive antimicrobial infusions. Insured patients may be discharged early to complete their antimicrobial courses at home with contracted nursing assistance or in lower-cost nursing facilities, but uninsured patients usually remain in the hospital, posing a burden particularly on safety-net hospitals [4,5].

Outpatient parenteral antimicrobial therapy (OPAT), defined as the administration of parenteral antimicrobial therapy for at least two doses on different days without intervening hospitalization [6,7], has gained wide acceptance in modern medical practice in the US [7,8]. The primary goal of an effective OPAT program is to allow patients to safely and effectively complete a planned treatment course in their home or alternate outpatient site [9]. Secondary goals of an OPAT program include avoiding the inconveniences, complications, and expense of hospitalization to complete a prescribed intravenous (IV) antibiotic course.

OPAT is generally delivered in one of the following four settings: an infusion center, a nursing home, at home with daily skilled nursing visits, or at home with self- or family-administered therapy [7]. The first three—healthcare-administered OPAT (H-OPAT)—are most commonly seen in the US and are achievable with adequate insurance funding. The fourth—self-administered OPAT (S-OPAT)—is relatively uncommon globally, with the few published studies having been conducted in England and Ireland [10–12].

Parkland Hospital is an 800-bed safety-net hospital serving the low-income patient population of Dallas County, Texas. An internal review of early efforts to treat medically stable patients requiring long courses of antibiotics outside the hospital found emergency department visits in >50% of these patients and a substantial 30-d readmission rate in May 2009. With multidisciplinary input from physicians, nurses, pharmacists, and case managers, we established an S-OPAT clinic to address this problem by centralizing services for patients discharged with S-OPAT (S1 Fig). Finding no published baseline experience in the US against which to evaluate our S-OPAT program, we undertook this study to determine whether the low-income, mostly non-English-speaking patients in our S-OPAT program could administer their own parenteral antimicrobials at home by gravity with the same or better outcomes than traditionally accepted models of outpatient care available to patients with funding for healthcare services (H-OPAT).

## Methods

### Ethics Statement

The University of Texas Southwestern Medical Center’s Institutional Review Board approved study procedures and waived written informed consent.

### Study Design and Participating Communities

Ahead of data analysis, we hypothesized that patients in our S-OPAT program would have better clinical outcomes—because of the standardized training and weekly follow-up—than patients in the H-OPAT group, who received services of variable quality from numerous unregulated home health agencies and other sub-acute-care settings contracted by the hospital. As the primary test of this hypothesis, we identified from the Parkland Hospital electronic medical records all hospital readmissions within 30 d of discharge from the hospitalization episode in which the antimicrobial therapy was started for all patients in the S-OPAT and H-OPAT groups discharged in fiscal years 2010 (1 October 2009 to 30 September 2010) through 2013 (1 October 2012 to 30 September 2013). The fiscal year 2010 for the H-OPAT group also included patients discharged in the previous 9 mo (1 January 2009 to 30 September 2010) to achieve an adequate sample size. As a secondary test, we detected all deaths within 1 y of discharge in the two groups from the hospital’s electronic death records and by telephone follow-up of all patients not known to have died but whose electronic medical records showed no clinical contacts beyond 1 y from discharge. We excluded patients who required hospitalization for the duration of antimicrobial therapy because they were ineligible for OPAT by the set criteria given in S2 Fig, e.g., unstable home environment (homeless), history of IV drug abuse, or need for continued medical care beyond antimicrobial infusions [13]. Accurate lists of patients in the S-OPAT and H-OPAT groups were obtained from the OPAT clinic log and the social work office that arranges home health services for funded patients. The completeness of the lists was verified by a search of the electronic medical records and pharmacy data for all patients prescribed IV antimicrobial agents at discharge. The infectious disease diagnosis requiring long-term IV administration of antimicrobial agents was obtained from the electronic medical records.

Since the allocation to S-OPAT or H-OPAT was largely determined by healthcare funding status, comparison of the outcomes was expected to be affected by selection bias and confounding. Consequently, we collected the following measures at the patient level to use in multivariable analyses: age, gender, race/ethnicity, language if not English-speaking, body mass index, healthcare funding source, type of infection requiring IV antimicrobial treatment, and comorbid diagnosis of diabetes mellitus and chronic renal insufficiency. These measures were collected from the patients’ electronic medical records. Race/ethnicity was derived from patient self-identification as documented in the electronic medical records. The census tract of each patient’s residential address was determined by the SAS Geocode procedure and by manual searching of online databases, and the following characteristics were obtained from the 2012 US census data [14]: home location (central city core, suburban, or rural), distance from patient’s home address to Parkland Hospital, and family income estimated from median income of the census tract. The treatment plans for all patients being discharged to receive long-term antibiotic therapy outside the hospital were routinely reviewed by an infectious disease pharmacy specialist, with the assistance of an infectious disease physician as needed, to ensure that all patients received therapy appropriate for their infections, i.e., therapy in accordance with written clinical guidelines such as those of the Infectious Diseases Society of America [9]. The selection of antibiotics was not constrained by financial considerations because we had low outpatient pricing under the federal 340B Drug Pricing Program for the care of low-income populations in public hospitals.

### Outpatient Services for the Healthcare-Administered OPAT Patients

Patients with healthcare funding (i.e., private health insurance, Medicare, or Medicaid) have several alternatives for long-term IV antimicrobial therapy outside the hospital. Those most often used in both the Parkland Hospital and private hospital settings are daily home visits by private nursing services or admission to skilled nursing facilities or sub-acute-care facilities. Patients discharged to care facilities are not necessarily more seriously ill than those discharged to home, but their insurance coverage designates care facility services as standard of care for outpatient antimicrobial therapy.

### Management Plan for the Self-Administered OPAT Patients

As stipulated in the OPAT protocol (S1 Fig), patients were accepted into the S-OPAT program according to written eligibility criteria (S2 Fig) applied through an infectious diseases/OPAT consultation [15]. Before hospital discharge, S-OPAT patients received standardized training in appropriate technique for self-administration of parenteral antimicrobial therapy delivered by counting the drops of antimicrobial-containing IV fluid delivered by gravity (no infusion pumps were provided). Education materials, developed in 2009–2010 at a fourth grade literacy level, included pictures of necessary supplies, hand hygiene technique, and technique for aseptically connecting antibiotic solution to the IV catheter (S3 and S4 Figs). Instruction was delivered verbally following a pamphlet printed in both English and Spanish. A smaller group of patients who spoke only less common languages—including Vietnamese (seven patients), the Burmese dialect of Chin (two patients), Ethiopian Amharic (three patients) and 12 additional languages/dialects—were also effectively trained with the assistance of the hospital’s multilingual telephone translation services.

Most importantly, following training, competency was established before discharge through a standardized protocol, developed by a multidisciplinary team of physicians, nurses, pharmacists, and case managers, requiring patients to repeatedly demonstrate mastery of all the steps in self-administration by gravity (S5 Fig). Patients were then followed throughout treatment with weekly clinic visits for maintenance of their peripherally inserted central catheter and laboratory monitoring [16], and evaluation by an infectious disease physician or nurse practitioner every 2 wk (S1 Fig). The monthly attendance rate for scheduled clinic follow-up visits averaged approximately 85%. The total number of days a patient required IV antimicrobial therapy as an outpatient that would have otherwise been administered in the inpatient setting was determined, to reflect the number of inpatient days saved by the S-OPAT program.

### Development of the Propensity Score

A propensity score [17,18] was developed by a stepwise logistic regression analysis to predict S-OPAT versus H-OPAT group membership based on all of the covariates listed in Table 1, using an entry criterion of *p* ≤ 0.05. Polychotomous nominal variables were included in the pool of predictors as sets of dummy variables. Ordinal measures were included as regression variables on the original scale as well as transformed by the log, square root, or square, and as sets of dummy variables. The final propensity score model (Table 2) classified group membership extremely well (area under the receiver operating characteristic curve = 0.91). Each patient’s probability of S-OPAT membership conditioned on the characteristics in the model calculated from the multivariable logistic regression model was added to the database as the propensity score, which was then arbitrarily categorized at quintiles, as is commonly done [17]. We compared the distribution of patients in the S-OPAT and H-OPAT groups across the quintiles of the propensity score to evaluate sample size adequacy within the higher quintiles (S1 Table). We found that excluding the top two quintiles from the final outcome analyses did not alter the findings.

**Table 1:** Baseline characteristics of the patients in the self-administered OPAT and healthcare-administered OPAT groups.

Characteristic	Distribution of Baseline Characteristic by Outpatient Antimicrobial Management		
	S-OPAT (*n* = 944)	H-OPAT (*n* = 224)	*p*-Value
**Age (years)**			<0.001
16–24	36 (3.8)	3 (1.3)	
25–44	266 (28.2)	33 (14.7)	
45–64	513 (54.3)	100 (44.6)	
≥65	129 (13.7)	88 (39.3)	
**Gender**			0.87
Male	583 (61.8)	137 (61.6)	
Female	361 (38.2)	87 (38.8)	
**Race/ethnicity**			<0.001
White non-Hispanic	213 (22.6)	73 (32.6)	
Hispanic	461 (48.8)	43 (19.2)	
Black non-Hispanic	236 (25.0)	100 (44.6)	
Other	34 (3.6)	8 (3.6)	
**Language**			<0.001
English only	599 (63.5)	197 (88.0)	
Spanish only	322 (34.1)	24 (10.7)	
Other language only	23 (2.4)	3 (1.3)	
**Home location**			<0.001
Central city core	900 (95.3)	198 (88.4)	
Suburban	19 (2.01)	11 (4.9)	
Rural	19 (2.01)	15 (6.7)	
Missing data	6 (0.6)	0 (0.0)	
**Healthcare funding source** *			<0.001
Medicare	168 (17.8)	129 (57.6)	
Medicaid	140 (14.8)	60 (26.8)	
Private insurance	61 (6.5)	15 (6.7)	
Charity	314 (33.3)	9 (4.0)	
Self-pay	261 (27.6)	11 (4.9)	
**Fiscal year of index hospital discharge** ^‡^			<0.001
2010	104 (11.0)	108 (48.2)	
2011	231 (24.5)	43 (19.2)	
2012	305 (32.3)	42 (18.8)	
2013	304 (32.2)	31 (13.8)	
**Body mass index (kg/m** ^**2**^ **)**			<0.001
Underweight (<18.5)	26(2.8)	17 (7.6)	
Normal (18.5–24.9)	210 (22.3)	47 (21.0)	
Overweight (25.0–29.9)	288 (30.5)	31 (13.8)	
Obese (≥30.0)	420 (44.5)	129 (57.6)	
**Type of infection requiring antimicrobial therapy**			<0.001
Bone and joint	405 (42.9)	53 (23.7)	
Bacteremia	148 (15.7)	33 (14.7)	
Skin and soft tissue	96 (10.2)	27 (12.1)	
Central nervous system	42 (4.5)	13 (5.8)	
Intra-abdominal	35 (3.7)	9 (4.0)	
Genitourinary	122 (12.9)	28 (12.5)	
Pulmonary/ENT	32 (3.4)	27 (12.1)	
Other type/site	64 (6.8)	35 (15.2)	
**Diabetes mellitus**			<0.001
Yes	195 (20.7)	19 (8.5)	
No	749 (79.3)	205 (91.5)	
**Chronic renal insufficiency**			<0.001
Yes	92 (9.8)	52 (23.2)	
No	852 (90.3)	172 (76.8)	

Data are given as *n* (percent).

*The Medicare group includes patients ≥65 y of age as well as younger patients with certain disabilities. Charity healthcare funding refers to care received through Dallas County’s assistance program for residents, Parkland Health Plus, which is provided to uninsured patients earning ≤200% of the federal poverty level; uninsured patients earning >200% of the federal poverty level must pay for their healthcare (self-pay).

^‡^Fiscal years run from 1 October to 30 September. For H-OPAT, fiscal year 2010 also includes the 9 mo before the fiscal year (1 January 2009 to 30 September 2009).

ENT, ear/nose/throat.

**Table 2:** Multivariable logistic regression model of propensity score for participation in the self-administered OPAT versus healthcare-administered OPAT program.

Variable	aOR	95% CI	*p*-Value*
**Healthcare funding source**			<0.001
Medicare	1.00 (ref)		
Medicaid	1.09	0.64–1.84	0.76
Private insurance	1.04	0.48–2.27	0.93
Charity	9.02	4.08–19.96	<0.001
Self-pay	11.07	5.15–23.81	<0.001
**Type of infection requiring antimicrobial therapy**			<0.001
Pulmonary/ENT	1.00 (ref)		
Bone and joint	9.29	3.93–21.96	<0.001
Bacteremia	7.51	2.92–19.32	<0.001
Skin and soft tissue	3.78	1.43–10.03	0.008
Central nervous system	2.80	0.91–8.65	0.07
Intra-abdominal	3.67	1.04–12.99	0.04
Genitourinary	4.00	1.52–10.52	0.005
Other type/site	3.04	1.16–8.00	0.024
**Age (years) (divided by 10)**	0.63	0.54–0.74	<0.001
**Home location**			<0.001
Suburban or rural	1.00 (ref)		
Central city core	6.06	2.92–12.57	<0.001
**Language**			<0.001
English	1.00 (ref)		
Spanish only	3.12	1.71–5.69	<0.001
Other language only	3.77	0.72–19.86	0.12
**Body mass index (kg/m** ^**2**^ **)**			<0.001
Underweight (<18.5)	1.00 (ref)		
Normal (18.5–24.9)	1.12	0.44–2.82	0.81
Overweight (25.0–29.9)	3.15	1.23–8.10	0.017
Obese (≥30.0)	1.02	0.43–2.42	0.96
**Fiscal year of index hospital discharge** ^‡^			<0.001
2010	0.07	0.04–0.13	<0.001
2011	0.51	0.28–0.93	0.03
2012	0.59	0.33–1.08	0.09
2013	1.00 (ref)		
**Diabetes mellitus**			<0.001
No	1.00 (ref)		
Yes	3.65	1.87–7.11	<0.001
**Chronic renal insufficiency**			<0.001
No	1.00 (ref)		
Yes	0.35	0.20–0.62	<0.001

The model’s area under the receiver operating characteristic curve was 0.91. Patients’ predicted probability of S- OPAT participation from the model is the propensity score used to control for selection bias in later outcome modeling.

*The *p*-values for the main category terms (e.g., health funding source) are from the type 3 analysis of the main effects of the nine categorical variables, and the *p*-values for the individual category terms test the difference between each category (e.g., Medicaid) and its referent category (indicated by aOR = 1.00; e.g., Medicare), all based on a sample size of 1,168 patients.

^‡^Fiscal years run from 1 October to 30 September. For H-OPAT, fiscal year 2010 also includes the 9 mo before the fiscal year (1 January 2009 to 30 September 2009).

aOR, adjusted odds ratio; ENT, ear/nose/throat; ref, referent category.

### Analyses of Outcomes

The primary study outcome was ≥1 readmission from all causes within 30 d of discharge, and the secondary outcome was death from all causes within 1 y of discharge. For each outcome, two models were developed. In model 1 the covariates included those characteristics in Table 1 that entered a stepwise proportional hazards analysis of time (in days) to readmission (or death) with *p* ≤ 0.05 as the entry criterion and with the indicator of S-OPAT versus H-OPAT forced in. Model 2 included the same covariates as model 1 except that, in addition, the propensity score was forced in as a categorical variable partitioned at quintiles, with the first quantile as the referent. The stability of model 2 was tested by repeating it three times with these three modifications [19]: (1) excluding from the analysis all patients in the fourth and fifth quintiles of the propensity score, (2) replacing the categorized propensity score with the continuous one as a quadratic effect in the model, and (3) using multiple logistic regression analysis in place of proportional hazards analysis. Variation in the result was tested with interaction terms for treatment group by infection type, year of index hospital discharge, race/ethnicity, and healthcare funding source. All analyses were performed with version 9.4 of SAS for Windows (SAS Institute), and all *p*-values are two-tailed, with p ≤ 0.05 considered statistically significant.

## Results

**Fig 1:**
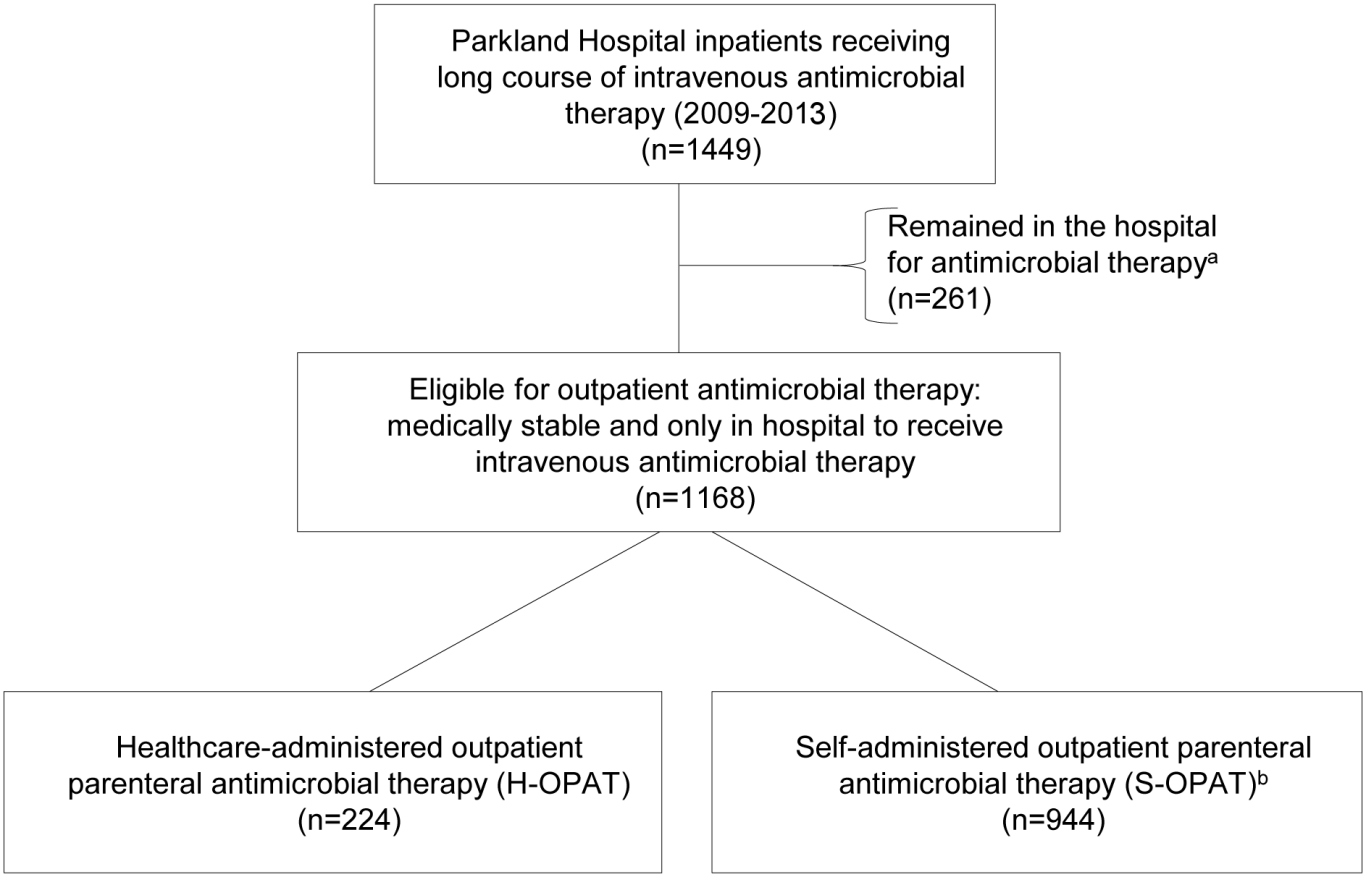
Summary of patient selection. ^a^Patients who were homeless, had a history of IV drug abuse, or were medically unstable. ^b^The eligibility criteria for inclusion in the S-OPAT group are given in S2 Fig.

This study includes 1,168 patients who were discharged from Parkland Hospital between 1 January 2009 and 30 September 2013 to receive IV antimicrobial therapy outside the hospital. The antimicrobial therapy of 944 (80%) of these patients was managed by the S-OPAT program, with weekly outpatient clinic follow-up, and the antimicrobial therapy of the remaining 224 (19%) of these patients was managed by a funded third party agency or institution (Fig 1). An additional 261 patients, with similar demographic characteristics but ineligible for outpatient infusion due to IV drug abuse, homelessness, or need for continued hospital care, remained in the hospital to complete their therapy and were excluded from the study (Fig 1).

### Baseline Group Differences

Compared with the H-OPAT group, the S-OPAT group had a higher percentage of patients with the following characteristics: healthcare funded by charity or self-pay, <65 y old, Hispanic, Spanish-only speaker, resident of the central city core, diabetic, and on long-term antimicrobial therapy for bone and joint infections, and a lower percentage of patients with chronic renal insufficiency (Table 1). Some S-OPAT patients had insurance funding, but their insurance plans did not extend to outpatient IV antimicrobial therapy (Table 1).

### 30-d All-Cause Readmission

The 30-d all-cause readmission rate was strongly associated with the 1-y all-cause mortality rate, the mortality rate for readmitted patients being 13.3% compared with 3.5% for those not readmitted (odds ratio, 4.3; 95% CI 2.5–7.3; *p <* 0.001; S1 Table), suggesting that complications in patients with potentially life-threatening infections that necessitate readmission are often very serious.

The 30-d all-cause readmission rate in the S-OPAT patients (16.7%) was 35% lower than that in the H-OPAT patients (23.7%) (crude hazard ratio, 0.65; 95% CI 0.47–0.88; *p* = 0.006; S1 Table), and this difference remained statistically significant after controlling for confounding in model 1 and for selection bias as well in model 2 (adjusted hazard ratio [aHR], 0.53; 95% CI 0.35–0.81; *p* = 0.003; Table 3). In model 2, the S-OPAT patients had lower readmission rates in each of the four years of the study (group-by-year interaction *p* = 0.31), in each of the infection type categories (group-by-infection-type interaction *p* = 0.99), in Hispanics versus non-Hispanics (group-by-race/ethnicity interaction *p* = 0.94), and in the self-pay group versus the other healthcare funding source groups (group-by-funding-source interaction *p* = 0.99). The repeat analysis with logistic regression gave similar results (Table 3). It is noteworthy that introducing the propensity score in model 2 yielded a lower hazard ratio than that in model 1.

**Table 3:** Multivariable proportional hazards regression models of 30-d readmission.

Variable	Model 1			Model 2		
	aHR	95% CI	*p*-Value*	aHR	95% CI	*p*-Value*
**Outpatient IV support**			0.002			0.003
H-OPAT	1.00 (ref)			1.00 (ref)		
S-OPAT	0.59^‡^	0.42–0.82	0.002	0.53^†^ ^‡^	0.35–0.81	0.003
**Healthcare funding source**			0.001			0.001
Medicare, private insurance, charity	1.00 (ref)			1.00 (ref)		
Self-pay	1.75	1.25–2.47	0.005	1.64	1.15–2.32	0.006
Medicaid	1.62	1.15–2.28	0.001	1.74	1.21–2.49	0.003
**Type of infection requiring IV antimicrobials**			0.001			0.001
Bone/joint, skin/soft tissue, intra-abdominal, genitourinary	1.00 (ref)			1.00 (ref)		
Bacteremia	1.43	1.03–1.99	0.05	1.43	1.03–1.99	0.03
Central nervous system	0.32	0.10–0.99	0.04	0.31	0.10–0.97	0.04
Pulmonary/ENT	0.43	0.19–0.98	0.05	0.44	0.19–1.02	0.06
Other type/site	0.55	0.30–0.99	0.003	0.54	0.29–0.98	0.04
**Chronic renal insufficiency**			0.003			0.003
No	1.00 (ref)			1.00 (ref)		
Yes	1.72	1.21–2.46	0.003	1.74	1.21–2.51	0.003
**Propensity score (quintiles)**						0.12
1				1.00 (ref)		
2				1.55	0.40–1.05	0.08
3				1.09	0.53–1.57	0.75
4				1.28	0.44–1.39	0.40
5				0.89	0.60–2.08	0.72

Model 1 controls for confounding with covariates; model 2 controls for confounding with covariates and for selection bias with the propensity score.

*The *p*-values for the main category terms are the effects from the type 3 tests, and those for the individual category terms are the maximum likelihood estimates, all based on a sample size of 1,168.

^‡^Replication of the two models with multiple logistic regression analysis gave similar results for all estimates; specifically, the odds ratio for S-OPAT was 0.59 (95% CI 0.40–0.86) in model 1 and 0.55 (95% CI 0.34–0.89) in model 2.

^†^Reanalysis after excluding patients in quintiles 4 and 5 of the categorical propensity score gave an aHR for S-OPAT of 0.51 (95% CI 0.33–0.79; *p* = 0.003). When the continuous propensity score was used in the model as a quadratic effect, the aHR for S-OPAT was 0.52 (95% CI 0.34–0.80; *p* = 0.003).

ENT, ear/nose/throat; ref, referent category.

Readmission for reasons directly attributable to antimicrobial infusions affected 21 of 944 (2.2%) S-OPAT patients and four of 224 (1.8%) H-OPAT patients. These included 13 with dysfunction of the peripherally inserted central catheter, ten whose underlying infection was not improving, six with renal or hepatic toxicity from the antibiotics, four with catheter-related bloodstream infection, and one with deep vein thrombosis. Readmission for reasons not directly related to the infusions occurred in 131 S-OPAT patients (13.9%) and 49 H-OPAT patients (21.9%).

### 1-y All-Cause Mortality

Despite the potentially life-threatening infections for which these patients were being treated, the 1-y all-cause mortality rate was low in both groups (S-OPAT, 5.4%; H-OPAT, 4.5%; *p* = 0.57; S1 Table). Stepwise proportional hazards analysis including the covariates and propensity score confirmed no significant difference in mortality between the treatment groups (aHR, 0.86; 95% CI, 0.37–2.00; *p* = 0.73; Table 4). The repeat analysis with logistic regression gave similar results (Table 4). Again, introducing the propensity score in model 2 yielded a lower hazard ratio for mortality than that in model 1.

**Table 4:** Multivariable proportional hazards regression models of 1-y mortality.

Variable	Model 1			Model 2		
	aHR	95% CI	*p*-Value	aHR	95% CI	*p*-Value*
**Outpatient IV support**						0.73
H-OPAT	1.00 (ref)			1.00 (ref)		
S- OPAT	0.94^‡^	0.45–1.96	0.87	0.86^†^ ^‡^	0.37–2.00	
**Healthcare funding source**						<0.001
Medicare, Medicaid, private, charity	1.00 (ref)			1.00 (ref)		
Self-pay	4.23	2.47–7.23	<0.001	5.48	3.09–9.73	
**Race/ethnicity**						0.01
White, black, other	1.00 (ref)			1.00 (ref)		
Hispanic	1.69	1.00–2.85	0.05	1.94	1.14–3.31	
**Diabetes mellitus**						0.01
No	1.00 (ref)			1.00 (ref)		
Yes	0.06	0.01–0.46	0.006	0.08	0.01–0.60	
**Age**						0.002
<65 y	1.00 (ref)			1.00 (ref)		
≥65 y	2.71	1.56–4.71	<0.001	2.48	1.41–4.37	
**Propensity score (quintiles)**						0.008
1				1.00 (ref)		
2				2.47	0.15–1.08	0.07
3				1.31	0.26–2.28	0.63
4				1.20	0.27–2.58	0.75
5				0.44	0.59–8.70	0.24

Model 1 controls for confounding with covariates; model 2 controls and for confounding with covariates and for selection bias with the propensity score.

*The *p*-values for the main category terms are the effects from the type 3 tests, and those for the individual category terms are the maximum likelihood estimates, all based on a sample size of 1,168.

^‡^Replication of the two models with multiple logistic regression analysis gave similar results for all estimates; specifically, the odds ratio for S-OPAT was 1.09 (95% CI 0.50–2.39) for model 1 and 1.05 (95% CI 0.43–2.55) for model 2.

^†^Reanalysis after excluding patients in quintiles 4 and 5 of the propensity score gave an aHR for S-OPAT of 0.82 (95% CI 0.35–1.91; *p* = 0.64). When the continuous propensity score was used in the model as a quadratic effect, the aHR for S-OPAT was 0.91 (95% CI 0.40–2.03; *p* = 0.81).

ref, referent category.

However, the analysis identified an unanticipated strong association of mortality with self-pay healthcare funding status (uninsured patients earning >200% of the poverty level, the “working poor”). Compared with 31 deaths (3.6%) in the 865 patients with Medicare, Medicaid, private insurance, or charity care (Dallas County residents earning ≤200% of the federal poverty level receive full care through the assistance program Parkland Health Plus), 30 deaths (12.4%) occurred in the 242 self-pay patients (aHR 5.48, 95% CI 3.09–9.73; *p <* 0.001; Table 4).

### Impact on Hospital Resource Utilization

Over the study period, the 944 patients in the S-OPAT program administered their own outpatient antimicrobial infusions at home for a median 26 d, saving the hospital 27,666 patient-days of hospitalization and, by the last year, freeing up an average of 26 hospital beds each day to accommodate other patients requiring more intensive services (Table 5).

**Table 5:** Impact of the self-administered OPAT program on the hospital’s inpatient bed utilization.

Fiscal Year of Index Hospital Discharge	Number of S-OPAT Patients	Median Number of Days of Outpatient Therapy per Patient	Total Number of Days of Outpatient Therapy for All S-OPAT Patients*	Average Number of Inpatient Hospital Beds Avoided per Day
2010	104	17	2,211	6.1
2011	231	27	6,848	18.7
2012	305	27	9,112	24.9
2013	304	29	9,495	26.0
All years	944	26	27,666	

*Before the S-OPAT clinic was started, all of these days would have been spent in the hospital just to receive antimicrobial infusions.

## Discussion

Our findings demonstrate that—after controlling for confounding with covariates and leveling preexisting differences between the two treatment groups as measured by a propensity score—the risk of 30-d readmission was significantly lower in the S-OPAT group than in the H-OPAT group, and the risk of dying within 1 y of hospital discharge was not significantly different in the two groups. This suggests that S-OPAT was an acceptable model for a select group of uninsured, medically stable patients, many of whom spoke no English, to complete extended courses of IV antimicrobial therapy for a variety of infections at home in an urban, safety-net-hospital setting. A standardized multilingual training program requiring patients to demonstrate proficiency in management of their IV lines and antimicrobial administration, followed by weekly clinic visits, allowed this broad cross-section of patients to manage their own antimicrobial administration for prolonged periods with good results. Despite having a higher mix of disadvantaged patients, the S-OPAT group had 30-d readmission and 1-y mortality rates that were equivalent to, or better than, those of patients with healthcare funding to support commercial outpatient services such as visiting nurses, skilled nursing facilities, and sub-acute-care facilities. The structure of our S-OPAT program meets national guidelines [9] and has all the elements of the outpatient antimicrobial therapy bundle recently proposed by Muldoon et al. [8].

The advent of newer antimicrobials offers medically stable patients hospitalized for long-term IV antimicrobial administration greater opportunity for completing their treatment course outside the hospital [12,20]. This leads not only to greater patient satisfaction but also to a theoretical lowering of the risks of complications from prolonged hospitalizations such as nosocomial infection with antibiotic-resistant hospital pathogens and *Clostridium difficile* colitis. S-OPAT benefits hospitals by reducing length of stay. By allowing patients to manage their own antimicrobial therapy at home, the program avoided a total of 27,666 hospital days of relatively low intensity care for antimicrobial infusion over the 4-y study period and, by the end, was freeing up an average of 26 hospital beds per day.

The greatest potential weakness of our study was the high possibility of bias in testing the effects of the S-OPAT intervention with an observational study design. That a patient’s healthcare funding status is associated with many risk factors for bad outcomes could introduce selection bias and confounding. Besides including covariates in the multivariable outcome analyses to control for confounding and increase precision, we developed and included in the outcome models a propensity score to control for selection bias. A propensity score is derived by initially developing a multivariable logistic regression model predicting treatment group membership (S-OPAT versus H-OPAT) from a combination of patients’ demographic and clinical characteristics [18]. The probability of being in one of the groups (e.g., the S-OPAT group) is then calculated for each patient in the study from the logistic regression equation. In the later multivariable outcome analysis (of 30-d readmission or 1-y mortality), incorporating the propensity score in the model balances between the two treatment groups all the characteristics that were in the propensity score development model, as well as any unmeasured characteristics that are correlated with the included characteristics but not those that are uncorrelated with the included characteristics. In general, propensity scores that adequately control for selection bias can enable observational studies to “approximate randomized experiments” [21]. In our study, however, the marked differences in the characteristics of patients in the two groups raise concerns over whether the findings could be due to residual selection bias not completely controlled for by the propensity score.

We believe this concern is mitigated by the following features of the analysis. First, the initial logistic regression model that generated our propensity score contained the actual characteristics that determined assignment to the S-OPAT or H-OPAT groups, e.g., the patients’ healthcare funding status and administrative, demographic, and clinical characteristics [22]. Second, the multivariable logistic regression model predicted group assignment very accurately (area under the receiver operating characteristic curve, 0.91), and thus the resulting propensity score should have controlled for selection bias well in the outcome analyses. Third, in the findings, the significant difference in 30-d readmission rate between the two treatment groups (S-OPAT lower than H-OPAT), adjusted by potential confounding variables and the propensity score, remained after excluding the patients in the top two quintiles of the propensity score, where sparse numbers (S1 Table) could have allowed residual bias. Fourth, and most powerfully, the apparent direction of the selection bias from inherent differences between patients in the two treatment groups went in the opposite direction from the finding. That is, reducing the selection bias by introducing the propensity score in model 2 (Table 3) caused the difference in 30-d readmission rate to widen further (the aHR decreased from 0.59 [41% lower in the S-OPAT group] in model 1 to 0.53 [47% lower in the S-OPAT group] in model 2), indicating that the selection bias had been tending to obscure the difference. Thus, we would expect any residual selection bias to be causing further underestimation of the group difference. We observed the same effect of the propensity score in the outcome model of 1-y all-cause mortality (the aHR decreased from 0.94 [6% lower in the S-OPAT group] in model 1 to 0.86 [14% lower in the S-OPAT group] in model 2; Table 4).

Other limitations are that with only 61 deaths, the study was underpowered to find subtle group differences in the secondary outcome, 1-y all-cause mortality, and because of the diversity of services accessed by patients in the H-OPAT group, the exact practices of the various third party settings are unknown. Although the study was conducted in a single hospital, since Parkland Hospital shares many similarities with other public hospitals, the findings are likely to apply to other hospitals caring for large numbers of low-income, uninsured patients. Whether the model applies to private hospital settings and to classes of insured patients should be evaluated.

A most provocative incidental finding was the disproportionately high risk of death in self-pay patients (the “working poor”). Irrespective of the type of outpatient antimicrobial administration and other sources of confounding and selection bias, patients making too much income to qualify for Medicaid or county assistance but too little to afford private insurance had a much higher risk of 1-y all-cause mortality from serious infections (aHR, 5.48; 95% CI 3.09–9.73, *p* < 0.001), as previously found with sepsis [23], cancer [24,25], and trauma [26,27]. One possible explanation is that the need to pay full price for care may have caused these patients to delay returning to the hospital when they had life-threatening complications. Hispanic patients also had a higher risk of 1-y all-cause mortality, possibly reflecting reluctance to return for care due to undocumented resident status [28,29]. Interestingly, diabetic patients had a significantly lower mortality rate, possibly resulting from an aggressive program by Parkland Hospital to manage transition of care services for diabetics during the period of the study. Including covariates for these characteristics in the propensity score model and in the outcome models avoided bias from their effects, and we confirmed this by demonstrating the lack of interaction with the treatment group variable in the outcome models.

In making the business case for S-OPAT in the United Kingdom, the British Society for Antimicrobial Chemotherapy reflected, “The idea that IV antibiotics can be safely administered at home, by patients themselves, is one that some years ago may have caused gasps of horror amongst the medical fraternity” [30]. In an era when hospitals are actively trying to reduce the rate of 30-d readmission, particularly when this quality measure is tied to reimbursement, we have shown clinical outcomes for our S-OPAT program to be better than the standard model of reimbursed care for 30-d readmission, and no different than the standard model for 1-y all-cause mortality.

Our study did not address reasons for the S-OPAT program’s superior outcome, but we suggest that rigorous patient training and weekly clinic consultation may equip patients to administer infusions more consistently for their own well-being than for-profit clinical businesses do, and the weekly clinic visits during outpatient antibiotic administration may identify and manage developing problems before they progress to hospitalization.

We conclude that S-OPAT, a model at the intersection of patient-centered care [31] and the hospital-at-home movement [32], can be an acceptable model of treatment for uninsured, medically stable patients. By offering the choice of the home environment over a skilled nursing facility, and the freedom of scheduling infusions not available with scheduled home health services, our model may also be an attractive option for patients with funding and access to third party healthcare services. Since our model is not resource intensive, it may be readily replicated in a variety of settings. If reimbursed suitably by health insurers, the S-OPAT model for suitable patients could largely supplant third party services. Our findings thus have important implications for healthcare financing agencies and for improving resource utilization in safety-net hospitals and other resource-limited settings that care for uninsured patients.

## Supporting Information

S1 Fig Self-administered OPAT protocol. (PDF)

S2 Fig Guidelines for patient selection. (PDF)

S3 Fig Excerpt from the beginning of the English-language materials for training patients participating in the Parkland Hospital self-administered OPAT program. (PDF)

S4 Fig An excerpt from the Spanish-language translation of the patient training materials explaining the administration of IV fluids, illustrated by the diagram of an IV administration set. (PDF)

S5 Fig Steps in the procedure that patients follow to demonstrate competence to self-administer IV antimicrobial agents before beginning the self-administered OPAT program in their home. Nursing staff demonstrate each of the steps for an OPAT-eligible patient and then verify competency by having the patient satisfactorily perform three separate return demonstrations of all the steps. If the patient is unable or unwilling to perform self-administration, a family member designated to administer the antibiotic for the patient at home is trained and assessed for competency by the same method.
(PDF)

S1 Table Association of patient characteristics with outpatient antimicrobial management alternatives and with the two outcome measures. (DOCX)

S2 Table Distribution of patients in the propensity score quintiles by outpatient antimicrobial management group. (DOCX)

S1 Text STROBE checklist. (DOCX)

S2 Text Documentation of study design. (DOCX)

## Abbreviations

aHR: adjusted hazard ratio
H-OPAT: healthcare-administered outpatient parenteral antimicrobial therapy
IV: intravenous
OPAT: outpatient parenteral antimicrobial therapy
S-OPAT: self-administered outpatient parenteral antimicrobial therapy
