# Supplementary figures and images for: Self-Administered Outpatient Antimicrobial Infusion by Uninsured Patients Discharged from a Safety-Net Hospital: A Propensity-Score-Balanced Retrospective Cohort Study
Source: PLoS Med. 2015 Dec 15;12(12):e1001922. doi: 10.1371/journal.pmed.1001922 (PMC4686020; doi:10.1371/journal.pmed.1001922)

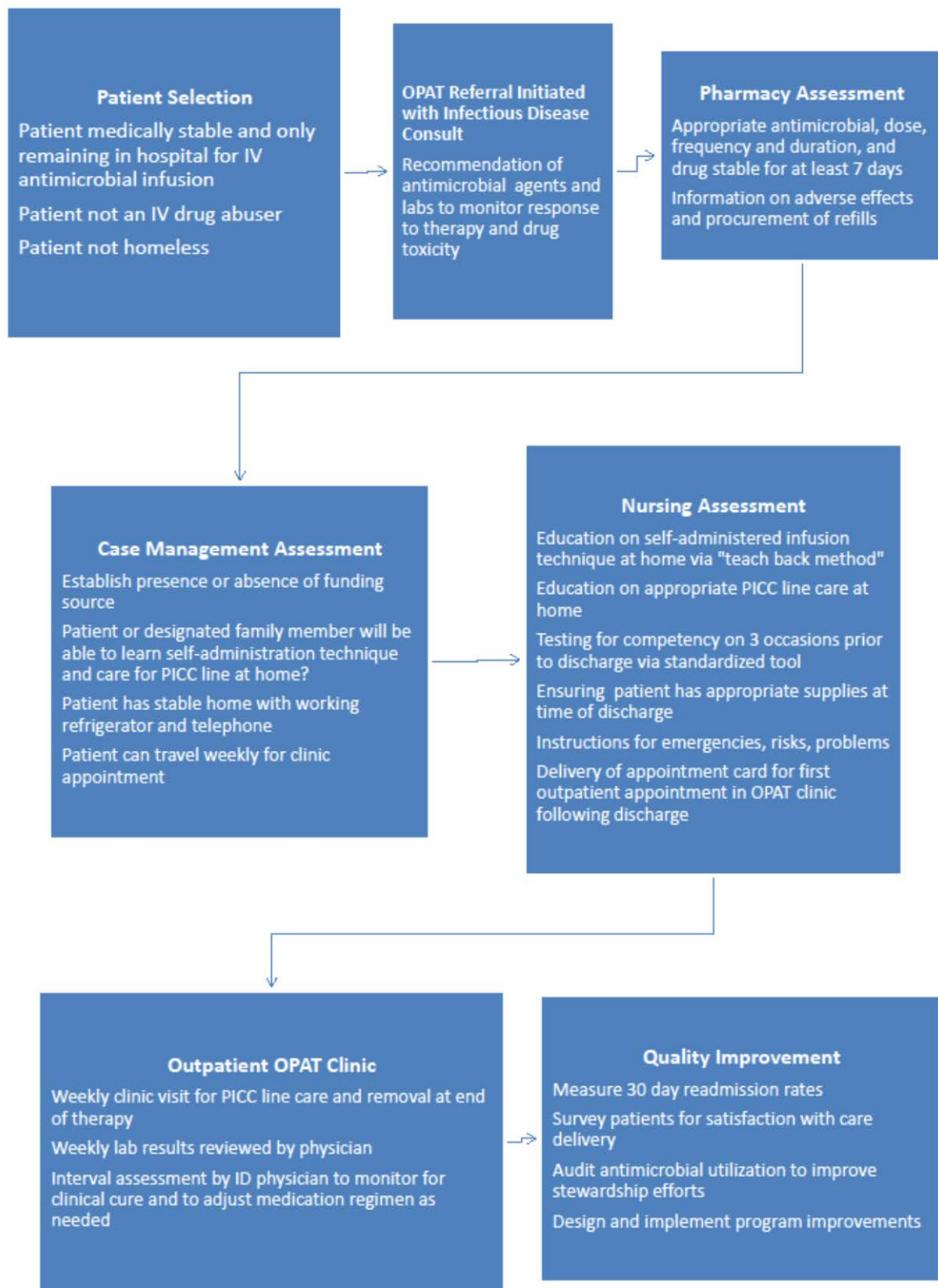

**Figure S1.** Self-administered OPAT protocol

Supplement: S1 Fig — (PDF) [file pmed.1001922.s001.pdf]
