# Supplementary material for: Self-Administered Outpatient Antimicrobial Infusion by Uninsured Patients Discharged from a Safety-Net Hospital: A Propensity-Score-Balanced Retrospective Cohort Study
Source: PLoS Med. 2015 Dec 15;12(12):e1001922. doi: 10.1371/journal.pmed.1001922 (PMC4686020; doi:10.1371/journal.pmed.1001922)
Supplement: S2 Fig — (PDF) [file pmed.1001922.s002.pdf]

1. The patient's medical condition is stable and the physician and nurse believe that the patient does not require hospitalization.
2. The patient and/or caregiver are capable of safely and effectively delivering parenteral antimicrobials (through return demonstration).
3. The patient will be living in a home environment that is safe and adequate to support care with good hygiene and safe/proper storage of supplies/medication. There are no psychosocial factors present which would preclude effective therapy in the home setting.
4. The patient and/or caregiver are aware of the risks of outpatient parenteral therapy.
5. The patient is not currently using illicit drugs or excessive alcohol and the patient is not at risk for using an intravenous catheter for administration of illicit drugs (this includes use of illicit drugs immediately prior to the acute presentation).
6. A working telephone is available to ensure communication between patient and health care provider.
7. The patient has transportation available to keep appointments with the health care provider.
8. The patient has a functioning intravenous catheter, which is appropriate for the duration of therapy ordered.
9. Only patients discharged from Parkland or patients whose parenteral antibiotic therapy was initiated by a Parkland physician are eligible for follow up in the OPAT clinic. Patients whose therapy was initiated at another hospital are not eligible (regardless of funding); the institution initiating outpatient antibiotic therapy is responsible for arranging and providing outpatient therapy until the course of treatment is completed.

Figure S2. Guidelines for patient selection
