# Supplementary material for: Self-Administered Outpatient Antimicrobial Infusion by Uninsured Patients Discharged from a Safety-Net Hospital: A Propensity-Score-Balanced Retrospective Cohort Study
Source: PLoS Med. 2015 Dec 15;12(12):e1001922. doi: 10.1371/journal.pmed.1001922 (PMC4686020; doi:10.1371/journal.pmed.1001922)
Supplement: S3 Fig — (PDF) [file pmed.1001922.s003.pdf]

Giving Your IV (Intravenous) Antibiotics Through Your PICC Line At Home

Parkland
Patient Education

Your doctor wants you to have antibiotics through your PICC line at home. These antibiotics treat the infection in ______________________________. You will need to give yourself these antibiotics for __________________ weeks.

During this time, you will have appointments at the Parkland clinic. It is very important for you to come to these clinic appointments because this is when we will check your blood, and check to be sure you are getting the right amount of the antibiotic. We will also put a fresh, sterile (no germs) dressing over your PICC line 1 (one) time each week at your clinic appointment. Your nurse will check that there is no infection at the place where the tube goes into your body.

Your first appointment is:____.
If you cannot come to this appointment, call 214-590-5061 to make another appointment.

Getting ready to give your antibiotic through your PICC line:

1. Clean off a clean, dry, flat place with alcohol, to put your supplies on, or put clean, dry paper towels down before you put your supplies down.

2. The supplies you will need to give your antibiotic through your PICC line are:
   – IV medicine bag
   – IV tubing
   – IV tubing Extension set
   – The blue Microclave cap
   – 2 pairs of gloves
   – Alcohol pads

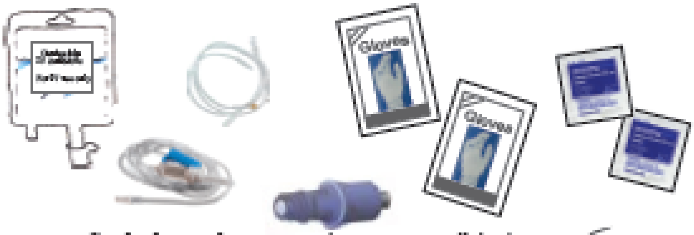

3. Always wash your hands before you flush the catheter, or give your antibiotics. Handwashing is **the most important** way to prevent infection!
   – Wash your hands with soap and water for 15 seconds. Then rinse and dry with a paper towel or clean cloth towel.
   – You can also use an alcohol hand rub instead of washing your hands.

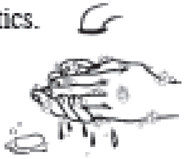

Figure S3. Excerpts from the beginning of the patient training materials for participation in the Parkland OPAT system
