# Supplementary material for: Self-Administered Outpatient Antimicrobial Infusion by Uninsured Patients Discharged from a Safety-Net Hospital: A Propensity-Score-Balanced Retrospective Cohort Study
Source: PLoS Med. 2015 Dec 15;12(12):e1001922. doi: 10.1371/journal.pmed.1001922 (PMC4686020; doi:10.1371/journal.pmed.1001922)
Supplement: S4 Fig — (PDF) [file pmed.1001922.s004.pdf]

______________________. Revise usted mismo y asegurarse de que está claro o transparente y que no tiene manchas o partículas flotando en él. Si no es claro, no lo use, tráigalo a la clínica en la próxima cita. Utilice otra bolsa que está claro.

Mantenga este antibiótico en el refrigerador? Sí o No

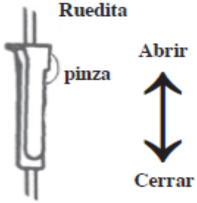

- Cierre la pinza en el tubo o línea y ponga la punta en el puerto en de salida de la bolsa intravenosa de antibióticos.
- Apriete la cámara de goteo para iniciar el flujo del antibiótico IV. Llene la cámara de goteo a la mitad.
- Cuelgue la bolsa a un nivel más alto que su cabeza.
- Abra lentamente la pinza para que el IV antibióticos llene el tubo o línea. Esto empuja todo el aire del tubo.
- Cierre la válvula con la ruedita o disco.

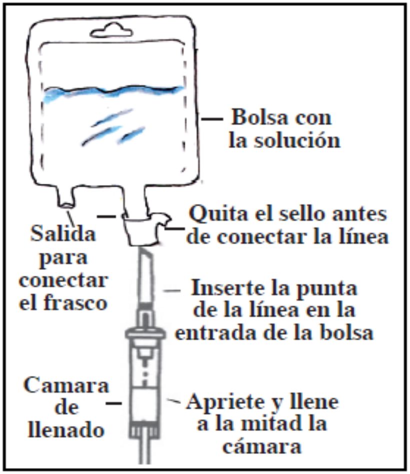

Recuerde - no deje que la punta del tubo toque nada.

El nombre de su antibiótico IV, que debe ser mezcla es: ______________________________________. Después de haberlo mezclado, verifique y asegurarse de que está claro o transparente y que no tiene manchas o partículas flotando en él. Si no está claro o transparente, no lo use. Tráigalo a la clínica en su próxima cita. Utilice otra bolsa que está claro.

Mantenga este antibiótico en el refrigerador? Sí o No

- Mezcle cada dosis al momento antes de usarlo.

(continúa)

Figure S4. An excerpt from the Spanish language translation of the patient training materials explaining administration of intravenous fluids, illustrated by the diagram of an intravenous administration set
