# Supplementary material for: Self-Administered Outpatient Antimicrobial Infusion by Uninsured Patients Discharged from a Safety-Net Hospital: A Propensity-Score-Balanced Retrospective Cohort Study
Source: PLoS Med. 2015 Dec 15;12(12):e1001922. doi: 10.1371/journal.pmed.1001922 (PMC4686020; doi:10.1371/journal.pmed.1001922)
Supplement: S5 Fig — Nursing staff demonstrate each of the steps for an OPAT-eligible patient and then verify competency by having the patient satisfactorily perform three separate return demonstrations of all the steps. If the patient is unable or unwilling to perform self-administration, a family member designated to administer the antibiotic for the patient at home is trained and assessed for competency by the same method. (PDF) [file pmed.1001922.s005.pdf]

State reason for IV antibiotics: “Treat infection in _______”
State length of treatment: weeks
State reason for clinic visits and frequency (“PICC dressing change, lab work; weekly”)
Locate phone number to order antibiotic (last page handout "MAR"; 214 590-8711 option "home antibiotics)
Clean flat surface with glass cleaner/alcohol and/or lay out clean paper towel for equipment
Identify equipment used: PICC line, IV tubing, extension set, adaptor, alcohol pad, IV med bag**
State why washing hands important when accessing line
Demonstrate proper hand-washing or use of alcohol hand rub**
Check the label on med bag (patient name, med name, exp. date)
Label IV tubing/check label on IV tubing; change every 3 days or if<br>spikes have touched anything
Check med: clear without anything in it
Close roller clamp of IV tubing
Take cover off outlet port of med bag
Spike bag with IV tubing without touching spike to anything
Squeeze the drip chamber until half full of liquid
Hang bag above patient’s head
Open the roller clamp and let the fluid fill the tubing
Close roller clamp of IV tubing and put on gloves
Clean PICC injection cap with alcohol**
Push 1 syringe of saline flush using a pulsating method**
Take cap of end of IV tubing without touching spike to anything (save cap); twist end of IV tubing into the injection cap**
Slowly open IV roller clamp and count drops per minute**
Take off gloves during infusion and put on clean gloves for disconnect
When IV complete, flush with 1 syringe saline flush<br>PICC with clamp: flush with 1 syringe of heparin once per day using<br>positive pressure
Disconnect tubing and replace cap over end of tubing
Document administration of med on MAR (fill in med name, date & time)
State what to do if there is blood in the injection cap (tighten, check for cracks, flush 1 time;

call if bleeding continues)
State reasons to call the OPAT Clinic for help:<br>PICC won't flush<br>Antibiotic won't run in<br>PICC dressing falls off
State reasons to go to Emergency Room:<br>PICC line is leaking, looks damaged, or coming out<br>Fever >101, redness, pain, swelling or drainage at PICC<br>insertion site
Patient to demonstrate how to protect PICC site from moisture (cover with dressing/plastic wrap tape down edges)
State two reasons you should always call 911: chest pain, shortness of breath, light headed

Figure S5. Steps in the procedure patients follow in demonstrating competence to self-administer intravenous antimicrobial agents before beginning the OPAT program in their home

Nursing staff demonstrate each of the above criteria for an OPAT eligible patient and then verify competency by having the patient satisfactorily perform 3 separate return demonstrations of all the steps. If the patient is unable or unwilling to perform self-administration, a family member designated to administer the antibiotic for the patient at home is trained and assessed for competency by the same method.
