# Supplementary material for: Self-Administered Outpatient Antimicrobial Infusion by Uninsured Patients Discharged from a Safety-Net Hospital: A Propensity-Score-Balanced Retrospective Cohort Study
Source: PLoS Med. 2015 Dec 15;12(12):e1001922. doi: 10.1371/journal.pmed.1001922 (PMC4686020; doi:10.1371/journal.pmed.1001922)
Supplement: S1 Table — (DOCX) [file pmed.1001922.s006.docx]

| **S1 Table*.* Association of patient characteristics with outpatient antimicrobial management alternative and the two outcome measures** | | | | | | | | | | |
| --- | --- | --- | --- | --- | --- | --- | --- | --- | --- | --- |
|  |  | **Distribution* of baseline characteristics by outpatient antimicrobial management** | | |  | **Rate† of readmission within 30 days of discharge** | |  | **Rate† of death within 1 year of discharge** | |
| **Characteristics** | | **S-OPAT** | **H-OPAT** | **P value** |  | **N/total (%)** | **P value** |  | **N/total** | **P value** |
| All | | 944 | 224 |  |  | 211/1168 (18.1) |  |  | 61/1168 (5.2) |  |
| Age (years) | |  |  | <0.001 |  |  | 0.45 |  |  | 0.017 |
| 16-24 | | 36 (3.8) | 3 (1.3) |  |  | 4/39 (10.3) |  |  | 0/39 (0.0) |  |
| 25-44 | | 266 (28.2) | 33 (14.7) |  |  | 58/299 (19.4) |  |  | 13/299 (4.35) |  |
| 45-64 | | 513 (54.3) | 100 (44.6) |  |  | 114/613 (18.6) |  |  | 28/613 (4.6) |  |
| ≥65 | | 129 (13.7) | 88 (39.3) |  |  | 35/217 (16.3) |  |  | 20/217 (9.2) |  |
| Gender | |  |  | 0.87 |  |  | 0.65 |  |  | 0.33 |
| Male | | 583 (61.8) | 137 (61.6) |  |  | 133/720 (18.5) |  |  | 34/720 (4.7) |  |
| Female | | 361 (38.2) | 87 (38.8) |  |  | 78/448 (17.4) |  |  | 27/448 (6.0) |  |
| Race/ethnicity | |  |  | <0.001 |  |  | 0.89 |  |  | 0.14 |
| White Non-Hispanic | | 213 (22.6) | 73 (32.6) |  |  | 56/286 (19.6) |  |  | 12/286 (4.2) |  |
| Hispanic | | 461 (48.8) | 43 (19.2) |  |  | 88/504 (17.5) |  |  | 35/504 (6.9) |  |
| Black Non-Hispanic | | 236 (25.0) | 100 (44.6) |  |  | 60/336 (17.9) |  |  | 12/336 (3.6) |  |
| Other | | 34 (3.6) | 8 (3.6) |  |  | 7/42 (16.7) |  |  | 2/42 (4.8) |  |
| Language | |  |  | <0.001 |  |  | 0.85 |  |  | 0.02 |
| English | | 599 (63.5) | 197 (88.0) |  |  | 147/796 (18.5) |  |  | 34/796 (4.3) |  |
| Spanish | | 322 (34.1) | 24 (10.7) |  |  | 60/346 (17.3) |  |  | 27/346 (7.8) |  |
| Other | | 23 (2.4) | 3 (1.3) |  |  | 4/26 (15.4) |  |  | 0/26 (0.0) |  |
| Urban-rural home location | |  |  | <0.001 |  |  | 0.63 |  |  | 0.56 |
| Central city core | | 900 (95.3) | 198 (88.4) |  |  | 195/1098 (17.8) |  |  | 57/1098 (5.2) |  |
| Suburban | | 19 (2.01) | 11 (4.9) |  |  | 7/30 (23.3) |  |  | 2/30 (6.7) |  |
| Rural | | 19 (2.01) | 15 (6.7) |  |  | 7/34 (20.6) |  |  | 1/34 (2.9) |  |
| Missing data | | 6 (0.6) | 0 (0.0) |  |  | 2/6 (33.3) |  |  | 1/6 (16.7) |  |
| Funding source‡ | |  |  | <0.001 |  |  | 0.002 |  |  | <0.001 |
| Medicare | | 168 (17.8) | 129 (57.6) |  |  | 55/297 (18.5) |  |  | 17/297 (5.7) |  |
| Medicaid | | 140 (14.8) | 60 (26.8) |  |  | 50/200 (25.0) |  |  | 6/200 (3.0) |  |
| Private insurance | | 61 (6.5) | 15 (6.7) |  |  | 12/76 (15.8) |  |  | 1/76 (1.3) |  |
| Charity | | 314 (33.3) | 9 (4.0) |  |  | 38/323 (11.8) |  |  | 7/323 (2.2) |  |
| Self-pay | | 261 (27.6) | 11 (4.9) |  |  | 56/272 (20.6) |  |  | 30/272 (11.0) |  |
| Fiscal year of index hospital discharge | |  |  | <0.001 |  |  | 0.70 |  |  | 0.04 |
| 2010* | | 104 (11.0) | 108 (48.2) |  |  | 40/212 (18.9) |  |  | 7/212 (3.3) |  |
| 2011 | | 231 (24.5) | 43 (19.2) |  |  | 43/274 (15.7) |  |  | 12/274 (4.4) |  |
| 2012 | | 305 (32.3) | 42 (18.8) |  |  | 64/347 (18.4) |  |  | 28/347 (8.1) |  |
| 2013 | | 304 (32.2) | 31 (13.8) |  |  | 64/335 (19.1) |  |  | 14/335 (4.2) |  |
| Body mass index (kg/m^2^) | |  |  | <0.001 |  |  | 0.68 |  |  | 0.17 |
| Underweight (<18.5) | | 26(2.8) | 17 (7.6) |  |  | 8/43 (18.6) |  |  | 4/43 (9.3) |  |
| Normal (18.5-24.9) | | 210 (22.3) | 47 (21.0) |  |  | 51/257 (19.8) |  |  | 14/257 (5.5) |  |
| Overweight (25.0-29.9) | | 288 (30.5) | 31 (13.8) |  |  | 51/319 (16.0) |  |  | 10/319 (3.1) |  |
| Obese (≥30.0) | | 420 (44.5) | 129 (57.6) |  |  | 101/549 (18.4) |  |  | 33/549 (6.0) |  |
| Infection requiring intravenous antimicrobials | |  |  | <0.001 |  |  | 0.006 |  |  | 0.006 |
| Bone and joint | | 405 (42.9) | 53 (23.7) |  |  | 83/458 (18.1) |  |  | 14/458 (3.1) |  |
| Bacteremia | | 148 (15.7) | 33 (14.7) |  |  | 48/181 (26.5) |  |  | 13/181 (7.2) |  |
| Skin and soft tissue | | 96 (10.2) | 27 (12.1) |  |  | 21/123 (17.1) |  |  | 9/123 (7.3) |  |
| Central nervous system | | 42 (4.5) | 13 (5.8) |  |  | 3/55 (5.5) |  |  | 1/55 (1.8) |  |
| Intra-abdominal | | 35 (3.7) | 9 (4.0) |  |  | 8/44 (18.2) |  |  | 6/44 (13.6) |  |
| Genitourinary | | 122 (12.9) | 28 (12.5 |  |  | 30/150 (20.0) |  |  | 11/150 (7.3) |  |
| Pulmonary/ENT | | 32 (3.4) | 27 (12.1) |  |  | 6/59 (10.2) |  |  | 0/59 (0.0) |  |
| Other site | | 64 (6.8) | 35 (15.2) |  |  | 12/98 (12.2) |  |  | 7/98 (7.1) |  |
| Diabetes mellitus | |  |  | <0.001 |  |  | 0.36 |  |  | <0.001 |
| Yes | | 195 (20.7) | 19 (8.5) |  |  | 34/214 (15.9) |  |  | 1/214 (0.5) |  |
| No | | 749 (79.3) | 205 (91.5) |  |  | 177/954 (18.6) |  |  | 60/954 (6.3) |  |
| Chronic renal insufficiency | |  |  | <0.001 |  |  | <0.001 |  |  | 0.16 |
| Yes | | 92 (9.8) | 52 (23.2) |  |  | 41/144 (28.5) |  |  | 11/144 (7.6) |  |
| No | | 852 (90.3) | 172 (76.8) |  |  | 170/1024 (16.6) |  |  | 50/1024 (4.9) |  |
| Outpatient IV support | |  |  | -- |  |  | 0.016 |  |  | 0.57 |
| H-OPAT | | -- | -- |  |  | 158/944 (16.7) |  |  | 51/944 (5.4) |  |
| S-OPAT | | -- | -- |  |  | 53/224 (23.7) |  |  | 10/224 (4.5) |  |
| Readmitted within 30 days of hospital discharge | |  |  | -- |  |  | -- |  |  | <0.001 |
| Yes | | -- | -- |  |  | -- |  |  | 28/211 (13.3) |  |
| No | | -- | -- |  |  | -- |  |  | 33/957 (3.5) |  |
| S-OPAT = Self-administered outpatient antibiotic therapy; H-OPAT = Healthcare-administered outpatient antibiotic therapy. | | | | | | | | | | |
| *Column percentages. †Row percentages. | | | | | | | | | | |
| ‡The Medicare group includes patients ≥65 years of age as well as younger ones with certain disabilities. Charity care is provided to uninsured patients earning ≤200% of the federal poverty level receiving full care through Dallas County’s residents assistance program Parkland Health Plus; whereas, uninsured patients earning >200% of the federal poverty level must pay for their healthcare (self-pay). | | | | | | | | | | |
|  | |  |  |  |  |  |  |  |  |  |
