# Supplementary material for: Self-Administered Outpatient Antimicrobial Infusion by Uninsured Patients Discharged from a Safety-Net Hospital: A Propensity-Score-Balanced Retrospective Cohort Study
Source: PLoS Med. 2015 Dec 15;12(12):e1001922. doi: 10.1371/journal.pmed.1001922 (PMC4686020; doi:10.1371/journal.pmed.1001922)
Supplement: S2 Table — (DOCX) [file pmed.1001922.s007.docx]

| **S2 Table. Distribution of patients on propensity score categories by outpatient antimicrobial management group** | | |
| --- | --- | --- |
| **Propensity score, categorized at quintiles** | **Outpatient antimicrobial management group** | |
|  | **S-OPAT** | **H-OPAT** |
| 1 | 73 | 160 |
| 2 | 193 | 41 |
| 3 | 216 | 18 |
| 4* | 230 | 4 |
| 5* | 232 | 1 |
| *Since the numbers of H-OPAT patients in categories 4 and 5 of the propensity score are small, the outcome analyses were validated by rerunning them after excluding the patients in these 2 propensity score categories. See the results in footnotes to Tables 3 and 4. | | |
