# Supplementary material for: Self-Administered Outpatient Antimicrobial Infusion by Uninsured Patients Discharged from a Safety-Net Hospital: A Propensity-Score-Balanced Retrospective Cohort Study
Source: PLoS Med. 2015 Dec 15;12(12):e1001922. doi: 10.1371/journal.pmed.1001922 (PMC4686020; doi:10.1371/journal.pmed.1001922)
Supplement: S1 Text — (DOCX) [file pmed.1001922.s008.docx]

STROBE Statement—checklist of items that should be included in reports of observational studies

|  | Item No. | Recommendation | Page  No. | Relevant text from manuscript |
| --- | --- | --- | --- | --- |
| **Title and abstract** | 1 | (*a*) Indicate the study’s design with a commonly used term in the title or the abstract | 2 | Methods & Findings section |
|  |  | (*b*) Provide in the abstract an informative and balanced summary of what was done and what was found | 2-3 | Methods & Findings section |
| Introduction | | | |  |
| Background/rationale | 2 | Explain the scientific background and rationale for the investigation being reported | 4-5 | Paragraph 1-3 of Introduction |
| Objectives | 3 | State specific objectives, including any prespecified hypotheses | 5 | First line of Study Design section |
| Methods | | | |  |
| Study design | 4 | Present key elements of study design early in the paper | 5-7 | Paragraph 3 on p. 5 and  Paragraphs 1and 2 on p. 6-7 |
| Setting | 5 | Describe the setting, locations, and relevant dates, including periods of recruitment, exposure, follow-up, and data collection | 2,5,10 | Paragraph 2 of abstract on p. 2. Paragraph 1 on p. 5 and  Years in Table 1 on p. 10 |
| Participants | 6 | (*a*) *Cohort study*—Give the eligibility criteria, and the sources and methods of selection of participants. Describe methods of follow-up | 5-6 | Paragraph 3 on p. 5,  Paragraph 1 on p. 6, and  Appendix Figure S2 |
|  |  | (*b*) *Cohort study*—For matched studies, give matching criteria and number of exposed and unexposed | 9,14 | First paragraph of Results section on p. 14. Also Table 1 on p. 9 |
| Variables | 7 | Clearly define all outcomes, exposures, predictors, potential confounders, and effect modifiers. Give diagnostic criteria, if applicable | 6, 8 | Paragraph 1 & 2 on p. 6,  Last paragraph on p. 8, and  Tables 1 and 2 |
| Data sources/ measurement | 8* | For each variable of interest, give sources of data and details of methods of assessment (measurement). Describe comparability of assessment methods if there is more than one group | 6 | Paragraphs 1 & 2 on p. 6 |
| Bias | 9 | Describe any efforts to address potential sources of bias | 6, 8 | Paragraph 2 on p. 6, last paragraph on p. 8-9, Table 2,  middle paragraph on p. 13 |
| Study size | 10 | Explain how the study size was arrived at | 6 | Third line on top paragraph |

Continued on next page

| Quantitative variables | 11 | Explain how quantitative variables were handled in the analyses. If applicable, describe which groupings were chosen and why | 9 | Top paragraph on p. 9 |
| --- | --- | --- | --- | --- |
| Statistical methods | 12 | (*a*) Describe all statistical methods, including those used to control for confounding | 8-9, 14 | Last paragraph on p. 8 to p. 9, Table 2, and top paragraph on p. 14 |
|  |  | (*b*) Describe any methods used to examine subgroups and interactions | 15 | Paragraph on 30-day all-cause readmission |
|  |  | (*c*) Explain how missing data were addressed | N/A | No missing data except 6 patients on the Census data |
|  |  | (*d*) *Cohort study*—If applicable, explain how loss to follow-up was addressed | 6 | Paragraph 1 |
|  |  | (*e*) Describe any sensitivity analyses | 13 | Top paragraph on p. 13 |
| Results | | | | |
| Participants | 13* | (a) Report numbers of individuals at each stage of study—eg numbers potentially eligible, examined for eligibility, confirmed eligible, included in the study, completing follow-up, and analysed | 14 | First paragraph of Results section |
|  |  | (b) Give reasons for non-participation at each stage | 14 | First paragraph of Results section |
|  |  | (c) Consider use of a flow diagram | 15 | Figure 1 |
| Descriptive data | 14* | (a) Give characteristics of study participants (eg demographic, clinical, social) and information on exposures and potential confounders | 9-12 | Table 1 |
|  |  | (b) Indicate number of participants with missing data for each variable of interest | 9-12 | Table 1 |
|  |  | (c) *Cohort study*—Summarise follow-up time (eg, average and total amount) | 5-6 | Last paragraph on p. 5 through  middle of p. 6 |
| Outcome data | 15* | *Cohort study*—Report numbers of outcome events or summary measures over time | 11 | Bottom of Table 1 |
|  |  | *Case-control study—*Report numbers in each exposure category, or summary measures of exposure | N/A |  |
|  |  | *Cross-sectional study—*Report numbers of outcome events or summary measures | N/A |  |
| Main results | 16 | (*a*) Give unadjusted estimates and, if applicable, confounder-adjusted estimates and their precision (eg, 95% confidence interval). Make clear which confounders were adjusted for and why they were included | 16-18 | Tables 3 and 4 |
|  |  | (*b*) Report category boundaries when continuous variables were categorized | N/A | Quintiles used |
|  |  | (*c*) If relevant, consider translating estimates of relative risk into absolute risk for a meaningful time period | N/A |  |

Continued on next page

| Other analyses | 17 | Report other analyses done—eg analyses of subgroups and interactions, and sensitivity analyses | 15, 19 | Paragraph on 30-day all-cause readmission on p. 15, and Table 5 footnote |
| --- | --- | --- | --- | --- |
| Discussion | | | | |
| Key results | 18 | Summarise key results with reference to study objectives | 20-21 | First paragraph of Discussion |
| Limitations | 19 | Discuss limitations of the study, taking into account sources of potential bias or imprecision. Discuss both direction and magnitude of any potential bias | 21-22 | Second full paragraph on p. 21 through top of p. 22 |
| Interpretation | 20 | Give a cautious overall interpretation of results considering objectives, limitations, multiplicity of analyses, results from similar studies, and other relevant evidence | 21-22 | Second full paragraph on p. 21 through top of p. 21 |
| Generalisability | 21 | Discuss the generalisability (external validity) of the study results | 23 | First paragraph |
| Other information | |  | | |
| Funding | 22 | Give the source of funding and the role of the funders for the present study and, if applicable, for the original study on which the present article is based | 25 | No funding received |

*Give information separately for cases and controls in case-control studies and, if applicable, for exposed and unexposed groups in cohort and cross-sectional studies.

**Note:** An Explanation and Elaboration article discusses each checklist item and gives methodological background and published examples of transparent reporting. The STROBE checklist is best used in conjunction with this article (freely available on the Web sites of PLoS Medicine at http://www.plosmedicine.org/, Annals of Internal Medicine at http://www.annals.org/, and Epidemiology at http://www.epidem.com/). Information on the STROBE Initiative is available at www.strobe-statement.org.
