# Supplementary material for: Self-Administered Outpatient Antimicrobial Infusion by Uninsured Patients Discharged from a Safety-Net Hospital: A Propensity-Score-Balanced Retrospective Cohort Study
Source: PLoS Med. 2015 Dec 15;12(12):e1001922. doi: 10.1371/journal.pmed.1001922 (PMC4686020; doi:10.1371/journal.pmed.1001922)
Supplement: S2 Text — (DOCX) [file pmed.1001922.s009.docx]

**Documentation of the Study Design**

The initial request for project approval submitted to Parkland’s Office of Research Administration documents that the original objective of the study was to compare the 30-day readmission rates of patients in the S-OPAT and H-OPAT groups, which remained the primary study objective.

The draft outline for data requests needed to complete the study described the study design including the data to be requested, the hypothesis and groups to be compared, the development of the propensity score, the outcome analysis and the sources of data to be requested. The first installment of the database was received on 8/23/2013 initiating an 8 month database construction and cleaning, and the first analysis began on 4/14/2014.
